# DNA Damage Repair Status Predicts Opposite Clinical Prognosis Immunotherapy and Non-Immunotherapy in Hepatocellular Carcinoma

**DOI:** 10.3389/fimmu.2021.676922

**Published:** 2021-07-15

**Authors:** Yunfei Chen, Xu Wang, Xiaofan Deng, Yu Zhang, Rui Liao, Youzan Li, Hongji Yang, Kai Chen

**Affiliations:** ^1^ The Third Department of Hepatobiliary Surgery and Organ Transplant Center, Sichuan Provincial People’s Hospital, University of Electronic Science and Technology of China, Chengdu, China; ^2^ The Third Department of Hepatobiliary Surgery and Organ Transplant Center, Chinese Academy of Sciences Sichuan Translational Medicine Research Hospital, Chengdu, China; ^3^ No. 2 Ward of Hepatobiliary Surgery, Sichuan Provincial People’s Hospital, University of Electronic Science and Technology of China, Chengdu, China; ^4^ No. 2 Ward of Hepatobiliary Surgery, Chinese Academy of Sciences Sichuan Translational Medicine Research Hospital, Chengdu, China

**Keywords:** Immune checkpoint inhibitors, hepatocellular carcinoma, DNA damage repair, tumor microenvironment, immunotherapy

## Abstract

Immune checkpoint inhibitors(ICIs) that activate tumor-specific immune responses bring new hope for the treatment of hepatocellular carcinoma(HCC). However, there are still some problems, such as uncertain curative effects and low objective response rates, which limit the curative effect of immunotherapy. Therefore, it is an urgent problem to guide the use of ICIs in HCC based on molecular typing. We downloaded the The Cancer Genome Atlas-Liver hepatocellular carcinoma(TCGA-LIHC) and Mongolian-LIHC cohort. Unsupervised clustering was applied to the highly variable data regarding expression of DNA damage repair(DDR). The CIBERSORT was used to evaluate the proportions of immune cells. The connectivity map(CMap) and pRRophetic algorithms were used to predict the drug sensitivity. There were significant differences in DDR molecular subclasses in HCC(DDR1 and DDR2), and DDR1 patients had low expression of DDR-related genes, while DDR2 patients had high expression of DDR-related genes. Of the patients who received traditional treatment, DDR2 patients had significantly worse overall survival(OS) than DDR1 patients. In contrast, of the patients who received ICIs, DDR2 patients had significantly prolonged OS compared with DDR1 patients. Of the patients who received traditional treatment, patients with high DDR scores had worse OS than those with low DDR scores. However, the survival of patients with high DDR scores after receiving ICIs was significantly higher than that of patients with low DDR scores. The DDR scores of patients in the DDR2 group were significantly higher than those of patients in the DDR1 group. The tumor microenvironment(TME) of DDR2 patients was highly infiltrated by activated immune cells, immune checkpoint molecules and proinflammatory molecules and antigen presentation-related molecules. In this study, HCC patients were divided into the DDR1 and DDR2 group. Moreover, DDR status may serve as a potential biomarker to predict opposite clinical prognosis immunotherapy and non-immunotherapy in HCC.

## Introduction

Hepatocellular carcinoma (HCC) accounts for 75%~85% of primary liver cancer cases, ranking sixth among the most common cancers in the world and fourth in terms of cancer-related deaths (1). However, traditional treatment is not ideal for patients with advanced HCC (2). As an inflammation-related tumor, HCC features an immunosuppressive tumor microenvironment (TME) that can promote immune tolerance through various mechanisms. There have been a series of advances in immunotherapy, and immunotherapy that activates the tumor-specific immune response brings new hope for the treatment of HCC (3–5). However, there are still some problems, such as uncertain curative effects, low objective response rates (ORRs), many adverse reactions, and even drug resistance after initial patient response (6, 7). Therefore, how to use molecular typing to improve the immune microenvironment, modify the immune response of patients, and guide the choice of immunotherapy or combination therapy scheme to effectively improve the efficacy of immunotherapy is an urgent problem to be solved and a future direction for the development of accurate treatments for HCC.

Various HCC-related risk factors can cause DNA damage. If damaged DNA is not repaired correctly in time, it can lead to gene changes and genome instability, which are generally considered common features of human HCC. Dysfunction of the DNA damage repair (DDR) process is related to susceptibility to HCC, and this process is often enhanced in HCC, resulting in a poor anticancer treatment effect against HCC cells (8). Chemotherapy is one of the few choices for most patients with advanced HCC who do not need surgery, and HCC shows different degrees of drug resistance to most chemotherapy regimens; as such, few chemotherapy drugs are available for HCC (9, 10). Many conventional chemotherapy drugs produce effects by inducing DNA double-strand breaks. HCC cells counteract the DNA damage caused by chemotherapy drugs by strengthening their DDR ability, which often leads to chemotherapy resistance (11, 12).

Mutations in members of DDR pathways may affect the efficacy of immunotherapy. Alterations in DDR signaling pathway members can lead to genomic instability and increased mutation frequency. Mutations can be used as potential biomarkers for the efficacy of immunotherapy. High mutation loads are closely related to increases in neoantigen loads (NAL) and tumor-infiltrating lymphocytes (TILs) (13, 14). Among the possible biomarkers, mismatch repair deficiency (MMR-D), homologous recombination gene mutations and POLE mutations (which affect the DDR signaling pathway) play an important role in the efficacy of immune checkpoint inhibitors (ICIs). The main mechanism is that mutations in repair genes are related to increases in NAL, CD4+ and CD8+ TILs, and the expression of cytotoxicity-related genes, PD-1 and PD-L1 (15, 16). However, the molecular status of the DDR pathway, the activity of the DDR pathway and the efficacy of immunotherapy in HCC are not clear. Therefore, it is particularly important to explore the potential significance of DDR pathway molecular typing and DDR pathway activity in predicting response to immunotherapy or routine treatment in HCC.

## Methods

### HCC Cohort and Immunotherapy Cohort

**Figure 1:**
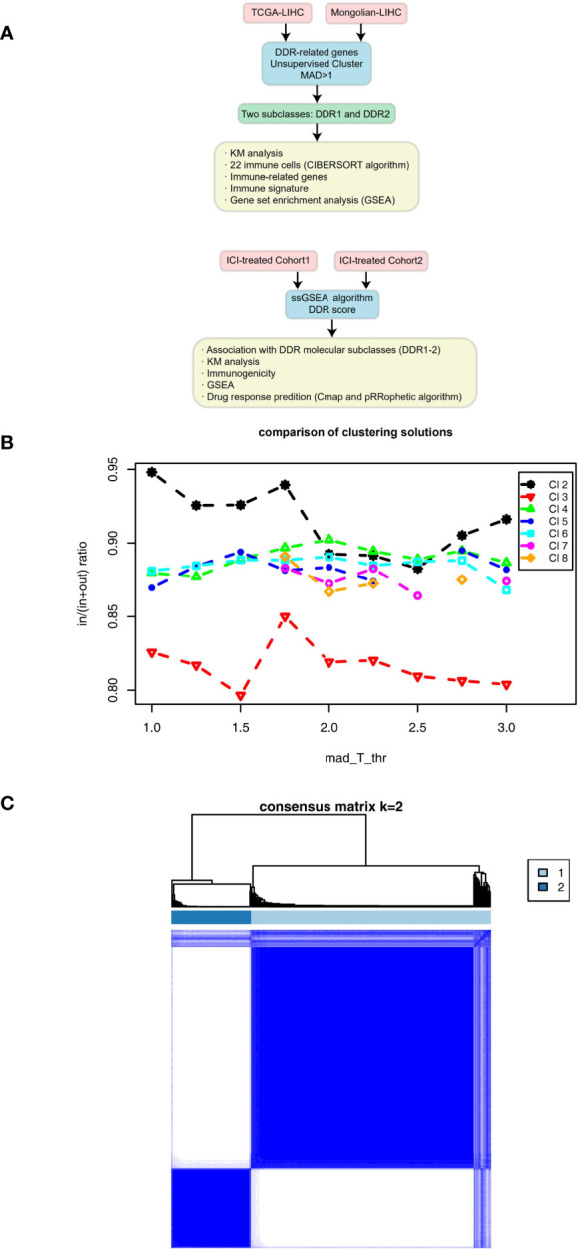
**(A)** Overview of the data processing in this study. **(B)** Mean coclustering ratio *vs* MAD threshold (in = mean pairwise coclustering within a cluster, out = mean pairwise coclustering across clusters), which was used as the objective function to find the optimal solution. **(C)** Coclustering matrix for a solution with 2 clusters and MAD threshold = 1.

We downloaded the The Cancer Genome Atlas-Liver hepatocellular carcinoma (TCGA-LIHC) cohort data, which includes mutation data, expression data and clinical data, from the TCGA database (https://portal.gdc.cancer.gov/) using the “TCGAbiolinks” R package (17). Additionally, we collected data from another LIHC cohort (Mongolian-LIHC, N = 70) (18), which included mutation data, expression data and clinical data, from published literature. We collected data from a bladder cancer cohort (ICI-treated BLCA, N = 348) receiving immunotherapy, which included mutation data, expression data and immunotherapy prognosis data (19), by using the IMvigor210CoreBiologies R package. Data from another melanoma cohort receiving ICIs were obtained from the Gene Expression Omnibus (GEO) database (GSE78220, N = 27) (20). These two immunotherapy cohorts were used to verify the potential utility of DDR typing for predicting immunotherapy response. See **Figure 1A** for the detailed analysis flow of this study.

### DDR Clustering and DDR Score Construction

The R package “ConsensusClusterPlus” was used to identify subtypes of DDR-related genes with highly variable expression (Median(-)> 1) in the TCGA-LIHC cohort, Mongolian-LIHC cohort and ICI-treated cohort (21). After unsupervised clustering (using the following parameters: maxK=8, reps=1,000, pItem=0.8, pFeature=0.8, clusterAlg=“km”, distance=“Euclidean”, innerLinkage=“average”, and finalLinkage=“average”), we obtained two types of DDR clusters. Then, we used the “limma” R package to analyze the differences in expression data in different DDR molecular subclasses in the TCGA-LIHC cohort, Mongolian-LIHC cohort and ICI-treated cohort (22). Single-sample gene set enrichment analysis (ssGSEA) algorithm and DDR-related gene sets were used to construct the DDR signature (23, 24). ssGSEA was similar to GSEA. For a given signature G of size N_G_ and single sample S, of the data set of N genes, the genes are replaced by their ranks according to their absolute expression from high to low: L = {r_1_,r_2_,…,r_N_}. An enrichment score ES(G,S) is obtained by a sum (integration) of the difference between a weighted ECDF of the genes in the signature PGw and the ECDF of the remaining genes P_NG_ (25):

$$\text{ES}(\text{G},\text{S})=\sum_{i=1}^{N}=[P_{G}^{w}(G,S,i)-P_{NG}(G,S,i)]$$

where $P_{G}^{w}(G,S,i)=\sum_{r_{j}\in G,j\leq i}\frac{|r_{j}|a}{\Sigma_{r_{j\in G}}|r_{j}|a}$

and ${\text{P}}_{\text{NG}}(\text{G},\text{S},\text{i})=\sum_{\text{r}\notin \text{G},\text{j}\leq \text{i}}\frac{1}{\left(\text{N}-{\text{N}}_{\text{G}}\right)}$

This calculation is repeated for each signature and each sample in the data set.

### Immune Correlation Analysis

The CIBERSORT algorithm (https://cibersort.stanford.edu/index.php) was used to evaluate the relative abundances of 22 immune cells in the TME of patients with LIHC (26). Immune-related genes and immune signatures were collected from published literature (27). The GSEA algorithm evaluates the difference in the enrichment degree of immune pathways, metabolic pathways and pathological pathways between different groups according to expression differences between the groups. Pathways with a P value less than 0.05 were considered to have statistically significant differences (28).

### Compound-Targeting Analysis

To identify which inhibitors/compounds may be useful for targeting cells with TP53 and RB1 co-mutations, we employed the Broad Institute’s connectivity map (CMap) build 02 (29), which is a publicly available online analytical tool (https://portals.broadinstitute.org/cmap/) that allows the analyzer to predict potential inhibitors/compounds based on upregulated and downregulated genes in a gene expression signature.

To further discover the mechanism of action (MoA) (30) and inhibitors/compounds, we used CMap tools (https://clue.io/). The CMap method is similar to GSEA, which can identify similarities and interactions (range: -1 to 1) based on differential gene expression data.

By using the pRRophetic R package (31), we constructed a ridge regression model based on the Genomics of Drug Sensitibity in Cancer (GDSC) cell line expression profile (www.cancerrxgene.org) (32) and the TCGA-LIHC, Mongolian-LIHC and ICI-treated cohort gene expression profiles to predict the half maximal inhibitory concentration (IC50) values of compounds/inhibitors.

### Statistical Analysis

For comparisons of factors such as immune cells and immune gene expression between the DDR1 and DDR2 groups, we used the Mann-Whitney U test. Fisher’s exact test and the chi-square test were used to analyze the contingency table. The Kaplan-Meier (KM) method and the log-rank test were applied in the survival analysis. When carrying out the survival analysis and comparing the efficacy of immunotherapy with that of traditional treatment, the survminer package (33) (surv_cutpoint function) was used to calculate the best cutoff for each cohort according to the relationship between the survival result and the ssGSEA score of DDR signaling. In this study, P < 0.05 was considered statistically significant, and all statistical tests were two-tailed. All statistical analyses and generation of visuals were performed using R software (version 3.6.3).

## Results

### Relationship Between DDR Type and Clinical Prognosis

**Figure 2:**
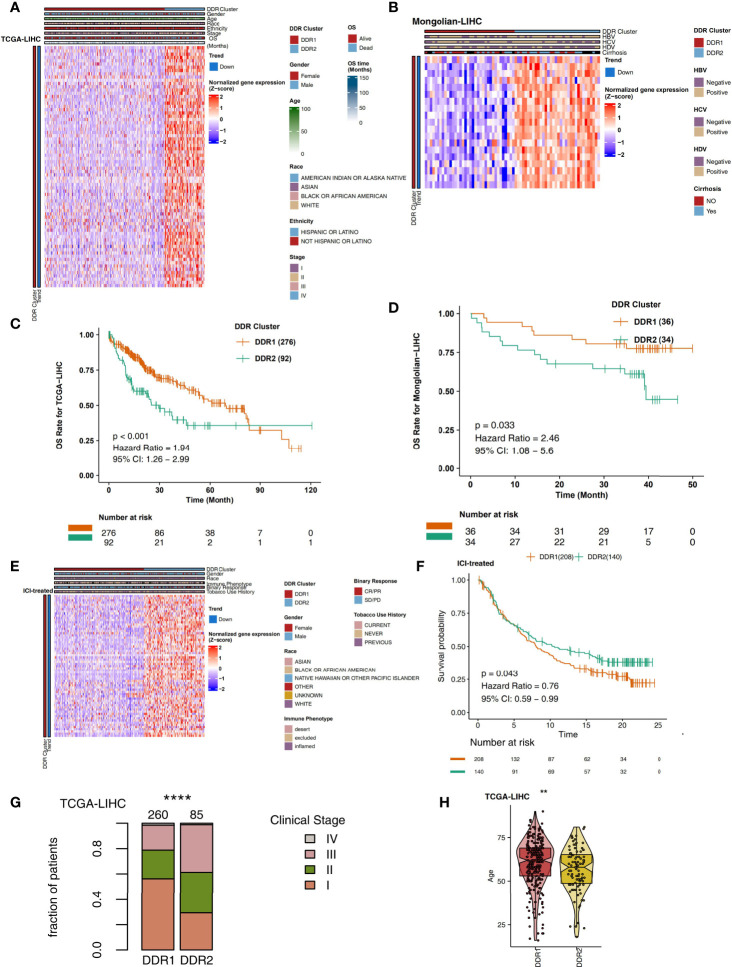
**(A)** Heatmap showing normalized expression levels of DDR-related signature genes (rows) across subjects (columns) classified into two molecular subclasses, DDR1 and DDR2, which were determined by an unsupervised approach (consensus clustering), in the TCGA-LIHC cohort. Each signature gene is significantly upregulated or downregulated in one subclass relative to the other subclasses, as indicated by the annotation bars on the left side. Clinical annotations are shown at the top. **(B)** Overall survival for subjects grouped according to molecular subclass (DDR1 and DDR2) in the TCGA-LIHC cohort. **(C)** Heatmap showing normalized expression levels of DDR-related signature genes (rows) across subjects (columns) classified into two molecular subclasses (DDR1 and DDR2), which were determined by an unsupervised approach (consensus clustering), in the Mongolian-LIHC cohort. Each signature gene is significantly upregulated or downregulated in one subclass relative to the other subclasses, as indicated by the annotation bars on the left side. Clinical annotations are shown at the top. **(D)** Overall survival for subjects grouped according to molecular subclass (DDR1 and DDR2) in the Mongolian-LIHC cohort. **(E)** Heatmap showing normalized expression levels of DDR-related signature genes (rows) across subjects (columns) classified into two molecular subclasses (DDR1 and DDR2), which were determined by an unsupervised approach (consensus clustering), in the ICI-treated BLCA cohort. Each signature gene is significantly upregulated or downregulated in one subclass relative to the other subclasses, as indicated by the annotation bars on the left side. Clinical annotations are shown at the top. **(F)** Overall survival for subjects grouped according to molecular subclass (DDR1 and DDR2) in the ICI-treated cohort. **(G)** Comparison of the proportions of patients in different clinical stages between two molecular subclasses (DDR1 and DDR2) in the TCGA-LIHC cohort. **(H)** Comparison of age between two molecular subclasses (DDR1 and DDR2) in the TCGA-LIHC cohort (**P < 0.01; ****P < 0.0001).

On the basis of the TCGA-LIHC expression data, we used the R package “ConsensusClusterPlus” to identify subtypes of samples based on the expression of DDR-related genes with highly variable expression (MAD) by unsupervised clustering (**Figure 1B**) and identified two main subtypes *via* heatmap analysis (**Figure 1C**). The cluster with low expression of DDR-related genes was called DDR1, while the cluster with high expression of DDR-related genes was called DDR2. The differential expression analysis of DDR-related genes between DDR1 and DDR2 in the TCGA-LIHC cohort showed that DDR1 had significantly lower expression of DDR-related genes than DDR2 (P < 0.05). The expression of DDR-related genes in the DDR2 group was significantly higher than that in the DDR1 group (P < 0.05; **Figure 2A**). The result of this typing was also verified in another cohort (Mongolian-LIHC), the samples of which were also divided into a DDR1 group with low expression of DDR-related genes and a DDR2 group with high expression of DDR-related genes (**Figure 2B**). In the TCGA-LIHC cohort receiving traditional treatment, the DDR2 group had a significantly shorter OS time than the DDR1 group (**Figure 2C**, P < 0.001, HR = 1.94; 95%CI: 1.26-2.99). In the Mongolian-LIHC cohort receiving traditional treatment, the DDR1 group had a shorter OS than the DDR2 group (**Figure 2D**; P = 0.033, HR = 2.46, 95%CI: 1.08-5.6). In a cohort of patients with advanced bladder cancer receiving immunotherapy, we were also able to divide the patients into two molecular subclasses: DDR1 patients with low expression of DDR-related genes and DDR2 patients with high expression of DDR-related genes (**Figure 2E**). Interestingly, among these ICI-treated patients, DDR2-type patients had significantly longer survival times from immunotherapy than DDR1-type patients (**Figure 2F**, P= 0.043, HR = 0.76, 95%CI: 0.59-0.99). To determine the associations between common clinical factors and DDR type, we compared the ages and clinical stages of patients in each DDR group. In the TCGA-LIHC cohort, DDR2 patients were significantly younger than DDR1 patients (**Figure 2G**, P < 0.05). In terms of clinical stage, we found that the DDR1 group had a higher proportion of early-stage patients (stage I and II) than the DDR2 group (**Figure 2H**, P < 0.05).

### Analysis of the Mutation Landscape in the Different DDR Groups

**Figure 3:**
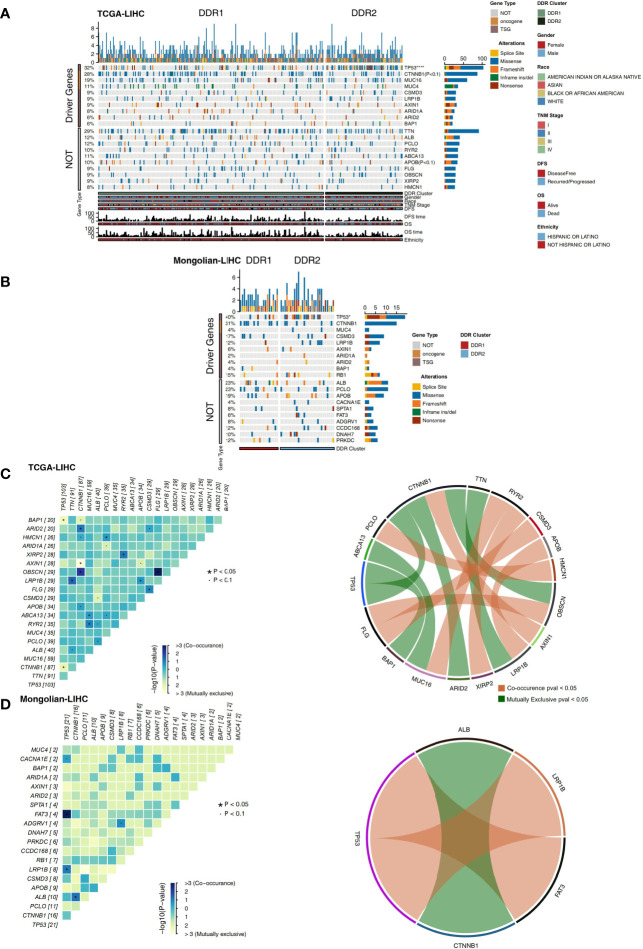
**(A)** Oncoplot showing mutated driver genes (rows) across subjects (columns) split into two molecular subclasses (DDR1 and DDR2) in the TCGA-LIHC cohort. The top panel shows the genes selected as the top 10 genes with driver mutations, while the bottom panel shows the top 10 additional genes with mutations that were not driver mutations. Clinical annotations are shown at the bottom. **(B)** Oncoplot showing mutated driver genes (rows) across subjects (columns) split into two molecular subclasses (DDR1 and DDR2) in the Mongolian-LIHC cohort. The top panel shows the genes selected as the top 10 genes with driver mutations, while the bottom panel shows the top 10 additional genes with mutations that were not driver mutations. Clinical annotations are shown at the bottom. **(C)** Heatmap showing mutually exclusive and cooccurring mutations in the genes from **(A)** in the TCGA-LIHC cohort. Ribbon plot showing cooccurrence or mutually exclusive relationships between pairs of genes from **(A)** in the TCGA-LIHC cohort. **(D)** Heatmap showing mutually exclusive and cooccurring mutations in the genes from **(B)** in the Mongolian-LIHC cohort. Ribbon plot showing cooccurrence or mutually exclusive relationships between pairs of genes from **(A)** in the Mongolian-LIHC cohort.

The nonsynonymous mutation data of TCGA-LIHC and Mongolian-LIHC were used to compare the mutations in the different DDR groups. The analysis of driving genes showed that in the TCGA-LIHC cohort, the DDR2 group had more patients with TP53 mutations than the DDR1 group, and the mutation frequencies of other driver mutations were not significantly different between the DDR1 and DDR2 groups (**Figure 3A**). Some nondriver mutations had high frequencies; the top 10 genes with the highest frequencies of driver mutations (Top10 genes) were TTN, ALB, PCLO, RYR2, ABCA13, APOB, FLG, OBSCN, and XIRP2 and HMCN1 (**Figure 3A**). Among these mutations, most were missense mutations. In the Mongolian-LIHC cohort, we found that patients in the DDR2 group had a higher mutation frequency of TP53 than patients in the DDR1 group. Similarly, the mutation frequencies of the Top10 genes were not significantly different between the DDR1 and DDR2 groups (**Figure 3B**). Then, we performed mutual exclusion/cooccurrence analysis on the mutations in each cohort. In the TCGA-LIHC cohort (**Figure 3C**), TP53 mutation was mutually exclusive with MUC16 or CTNNB1 mutation. In the Mongolian-LIHC cohort (**Figure 3D**), TP53 mutation and FAT3 or LRP1B mutation were cooccurring mutations. The above results suggest that there is no significant difference in the frequencies of high-frequency nonsynonymous mutations in driver genes between the with DDR1 and DDR2 groups.

### Analysis of the Immune Microenvironment in the Different DDR Types

**Figure 4:**
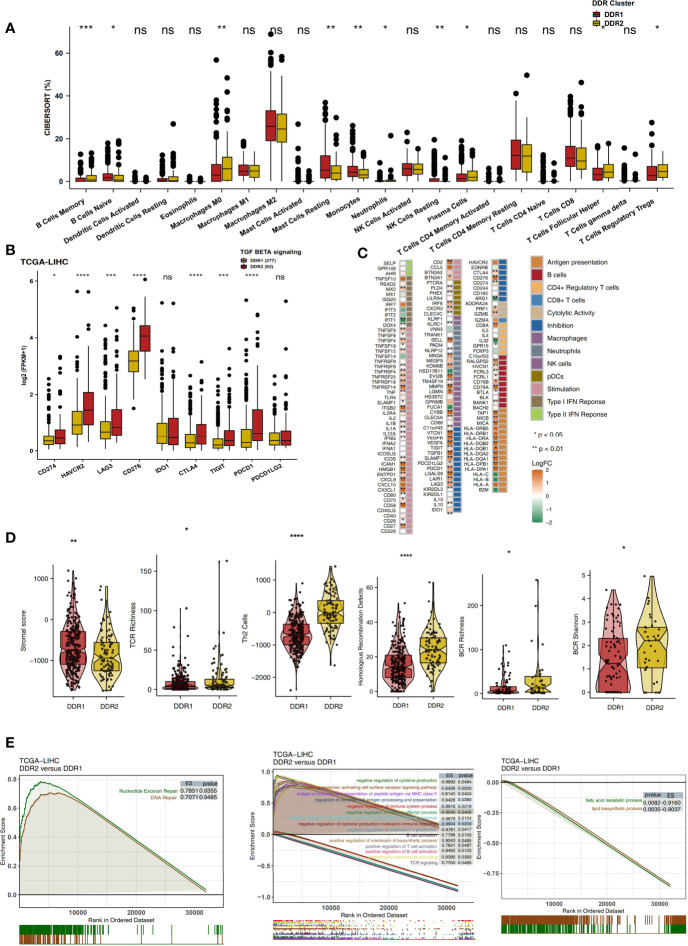
**(A)** Comparison of the proportions of 22 immune cells estimated by CIBERSORT between two molecular subclasses (DDR1 and DDR2) in the TCGA-LIHC cohort. **(B)** Comparison of the expression levels of immune checkpoint molecules between two molecular subclasses (DDR1 and DDR2) in the TCGA-LIHC cohort. **(C)** Heatmap depicting the mean differences in immune-related gene mRNA expression between two molecular subclasses (DDR1 and DDR2) in the TCGA-LIHC cohort. The y-axis indicates the gene names. Each square represents the fold change of or difference in each indicated immune-related gene between DDR1 and DDR2 tumors in the TCGA-LIHC cohort. Red indicates upregulation, while blue indicates downregulation. **(D)** Comparison of the immune signature scores between two molecular subclasses (DDR1 and DDR2) in the TCGA-LIHC cohort. **(E)** Biological function pathways, such as DNA repair, immune-related and immune-exhausted pathways, identified as enriched between DDR1 and DDR2 tumors in the TCGA-LIHC cohort. GSEA of hallmark gene sets downloaded from MSigDB. All transcripts were ranked by the log2 (fold change) value between DDR1 and DDR2 tumors. Each run was performed with 1,000 permutations. Pathways with significant enrichment between DDR1 and DDR2 tumors are shown. (ns, not significant; *P < 0.05; **P < 0.01; ***P < 0.001; ****P < 0.0001; Mann-Whitney U test).

The immune microenvironment is one of the key factors affecting the efficacy and clinical benefits of cancer patients receiving ICIs. Therefore, we explored the differences in the immune microenvironment in different DDR molecular subclasses in terms of the proportions of immune cells, the expression levels of immune-related genes, the immune status and the enrichment degree of specific pathways. CIBERSORT analysis showed that DDR2 patients had significantly increased proportions of activated immune cells (**Figure 4A**; P < 0.05), such as memory B cells, activated memory CD4+ T cells, M0 macrophages, plasma cells and T follicular helper cells (Tfhs). Checkpoint molecules are an important target of ICIs, and we analyzed the expression of checkpoint molecules in different DDR types. Compared with the DDR1 type, the DDR2 type had significantly increased expression of immune checkpoint molecules (**Figure 4B**; P < 0.05), such as CD274, HAVCR2, LAG3, CD276, CTLA4, TIGIT and PDCD1. Additionally, inflammatory factors and proteins with other immune functions (antigen presentation and other functions) also play a key role in the response to immunotherapy. The expression levels of genes related to antigen processing and presentation (HLA-DPA1, HLA-DPB1, HLA-DQA1, HLA-DQB1. HLA-DQB2, and HLA-DRA), chemokine genes (CX3CL1 and CXCL9) and proinflammatory molecule genes (TNFSF9, IL1B, IL1A, and IFNG) in the DDR2 group were significantly higher than the respective levels in of the DDR1 group (P < 0.05; **Figure 4C**). The result of immune signature analysis showed that the DDR2 group had significantly higher immune scores, indicating characteristics such as BCR richness, BCR Shannon diversity, TCR richness and Th2 cell features, than the DDR1 group (P < 0.05; **Figure 4D**). In contrast, the stromal score of patients in the DDR1 group was significantly higher than that of patients in the DDR2 group (P < 0.05; **Figure 4D**). In the TCGA-LIHC cohort, GSEA was used to analyze and compare the enrichment of pathways in DDR1 and DDR2 patients. For some DDR-related signaling pathways (such as the nucleotide excision repair pathway), immune-related pathways (such as pathways related to the positive regulation of interleukin-6 biosynthetic processes, B cell activation, the positive regulation of T cell activation, TCR signaling, antigen processing and the presentation of peptide antigen *via* MHC class II) were significantly activated in the DDR2 group compared with the DDR1 group. In contrast, the activities of some immune depletion-related pathways (such as pathways related to lipid biosynthetic processes and fatty acid metabolic processes) were significantly higher in the DDR1 group than in the DDR2 group (**Figure 4E**). Additionally, the above GSEA results were also verified in the Mongolian-LIHC cohort (**Figure S1**). We further demonstrated the differences in the expression levels of genes in the above pathways between the DDR1 and DDR2 groups *via* heatmap analysis (**Figures S2**, **S3**).

### Relationship Between DDR Score and Clinical Prognosis

**Figure 5:**
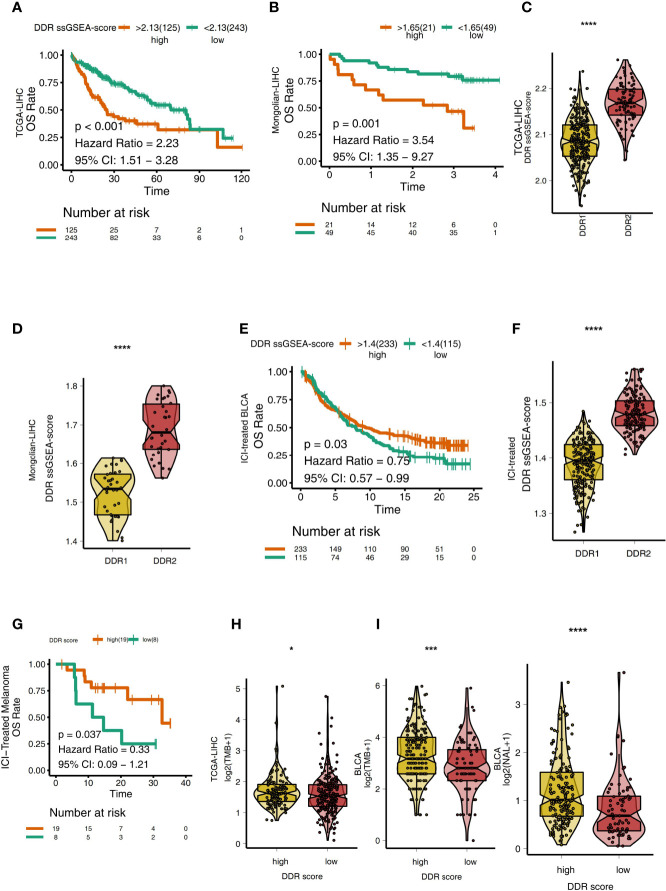
**(A)** Overall survival for subjects grouped according to DDR score subtype (high DDR score and low DDR score) in the TCGA-LIHC cohort. **(B)** Overall survival for subjects grouped according to DDR score subtype (high DDR score and low DDR score) in the Mongolian-LIHC cohort. **(C)** Comparison of the DDR scores between two molecular subclasses (DDR1 and DDR2) in the TCGA-LIHC cohort. **(D)** Comparison of the DDR scores between two molecular subclasses (DDR1 and DDR2) in the Mongolian-LIHC cohort. **(E)** Overall survival for subjects grouped according to DDR score subtype (high DDR score and low DDR score) in the ICI-treated BLCA cohort. **(F)** Comparison of the DDR scores between two molecular subclasses (DDR1 and DDR2) in the ICI-treated BLCA cohort. **(G)** Overall survival for subjects grouped according to DDR score subtype (high DDR score and low DDR score) in the ICI-treated melanoma cohort. **(H)** Comparison of TMB between two molecular subclasses (high DDR score and low DDR score) in the TCGA-LIHC cohort. **(I)** Comparison of TMB and NAL between two molecular subclasses (high DDR score and low DDR score) in the ICI-treated cohort. (*P < 0.05; ***P < 0.001; ****P < 0.0001; Mann-Whitney U test).

To explore the relationship between the DDR score and the prognosis of LIHC patients receiving traditional treatment, we determined the DDR score using the ssGSEA algorithm and DDR-related gene sets. In TCGA-LIHC, patients with higher DDR scores had significantly reduced OS compared with those with lower DDR scores (**Figure 5A**, P < 0.001, HR = 2.23, 95%CI: 1.51-3.28). Similarly, in the Mongolian-LIHC cohort, patients with high DDR scores had shorter OS than patients with low DDR scores (**Figure 5B**; P = 0.001, HR = 3.54, 95%CI: 1.35-9.27). Next, we analyzed the difference in DDR scores in different DDR groups. In both the TCGA-LIHC and Mongolian-LIHC cohorts, DDR2 patients had higher DDR scores than DDR1 patients (P < 0.0001, **Figures 5C, D**). This result was consistent with the analysis of DDR classification and clinical prognosis. To further verify the utility of the DDR score in patients receiving ICIs, we also determined the DDR scores in an ICI-treated BLCA cohort. In the ICI-treated BLCA cohort, patients with high DDR scores had significantly longer OS after immunotherapy than patients with low DDR scores (**Figure 5E**, P = 0.03, HR = 0.75, 95%CI: 0.57-0.99). Additionally, patients with the DDR2 type had higher DDR scores than patients with the DDR1 type (**Figure 5F**, P < 0.0001). In another ICI-treated melanoma cohort, compared with the survival time of patients with low DDR scores, the survival time of patients with high DDR scores was significantly prolonged (**Figure 5G**, P = 0.037, HR =0.33). In the TCGA-LIHC cohort, patients with high DDR scores had significantly higher TMB levels than those with low DDR scores (P < 0.05; **Figure 5H**). Similarly, in a BLCA cohort receiving immunotherapy, we also found that patients with high DDR scores had higher immunogenicity than patients with low DDR scores, and the patients with high DDR scores showed increased TMB and NAL levels (**Figure 5I**, all P < 0.05).

### Association Between the DDR Score and Sensitivity to Other Drugs

**Figure 6:**
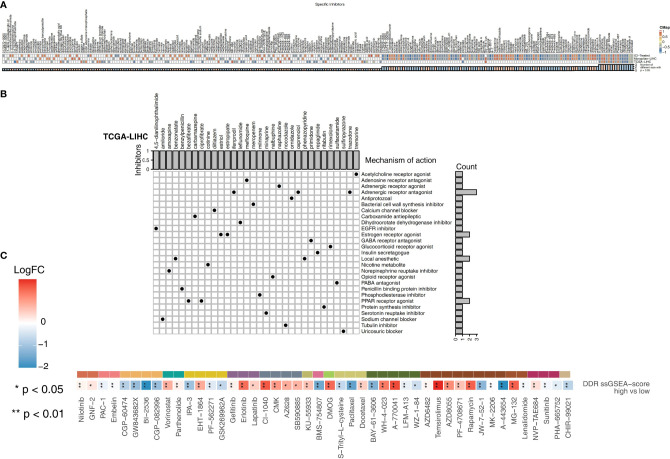
**(A)** Heatmap showing the enrichment scores (positive in blue and negative in red) of each compound from the CMap analysis of the TCGA-LIHC, Mongolian-LIHC and ICI-treated cohorts. The enrichment score (ES) ranged from -1 (negative connectivity) to 1 (positive connectivity). **(B)** Heatmap showing each compound from the CMap analysis that shares MoAs (rows) sorted by the number of compounds with shared MoAs in the remaining high DDR score and low DDR score tumors. **(C)** Heatmap depicting the mean differences in IC50 values between two molecular subclasses (high DDR score and low DDR score tumors) in the TCGA-LIHC cohort. The y-axis indicates the drug names. Each square represents the fold change of or difference in each indicated IC50 value between high DDR score and low DDR score tumors in the TCGA-LIHC cohort. Red indicates upregulation, while blue indicates downregulation. The drug types are shown on the left.

We used CMap analysis to predict therapeutic drugs and targets for the high DDR score group. CMap is a gene expression profile database containing gene expression data developed by the Broad Research Institute that is mainly used to reveal functional relationships between small molecule compounds, genes and disease states. The relationships between these factors are evaluated by a score, which ranges from -1 to 1. The results are arranged in descending order from high to low. The closer the value is to -1, the more likely the small molecules are to be an antagonist in patients with a high DDR score (**Figure 6A**). Therefore, these antagonistic small molecules can be candidate drugs for the treatment of patients with high DDR scores. We found that 8-azaguanine, bufexamac, estriol (an estrogen receptor agonist), oxetacaine, pyrvinium, repaglinide (an insulin secretagogue), rimexolone (a glucocorticoid receptor agonist) and trazodone (an adrenergic receptor antagonist) may be candidate drugs for treating patients with high DDR scores (**Figure 6B**). Additionally, we predicted the drug sensitivity of TCGA-LIHC patients by using the pRRophetic algorithm and a ridge regression model. Targeting the cell cycle (CGP-60474, GW 843682x, BI-2536, and CGP-082996), PI3K/mTOR signaling (JW-7-52-1, MK-2206, and A-443654), RTK signaling (sunitinib and PHA-665752) and WNT signaling (CHIR-99021) was significantly more effective in high DDR score patients than in low DDR score patients (**Figure 6C**).

### Differences in Pathway Activation Degree in the High and Low DDR Score Groups

**Figure 7:**
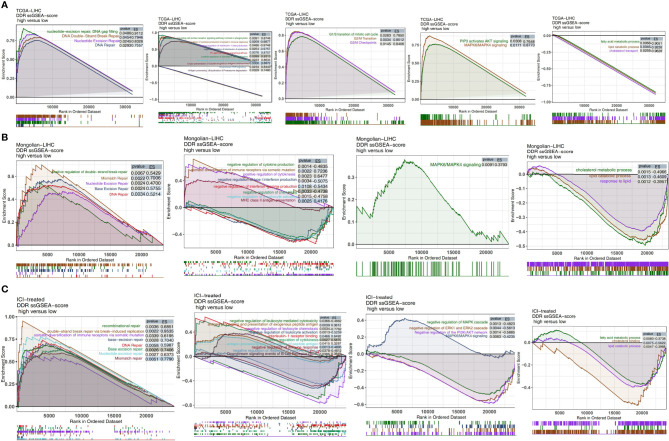
Biological function pathways, such as DNA repair, immune-related, cell cycle, drug resistance, and immune exhaustion pathways, identified as enriched between high DDR score and low DDR score tumors in the TCGA-LIHC cohort **(A)**, Mongolian-LIHC cohort **(B)** and ICI-treated cohort **(C)**.

In the TCGA-LIHC cohort (**Figure 7A**), we found that LIHC patients with high DDR scores showed significant activation of DNA repair-related signaling pathways (pathways related to nucleotide-excision repair, DNA gap filling, nucleotide excision repair, and DNA double-strand break repair), immune pathways (pathways related to downstream TCR signaling and positive regulation of activated T cell proliferation), cell cycle-related pathways (pathways related to G2/M checkpoints, the G2/M transition, and the G1/S transition of the mitotic cell cycle), and traditional drug resistance pathways (pathways related to MAPK6/MAPK4 signaling and PIP3, which activate AKT signaling) compared with patients with low DDR scores. In contrast, the activity of some pathways, such as pathways related to fatty acid metabolic processes, lipid catabolic processes and cholesterol transport, was significantly higher in patients with low DDR scores than in those with high DDR scores. In the Mongolian-LIHC cohort, the activity of DNA repair and immune-related pathways in the immune microenvironment of patients with higher DDR scores was significantly higher than that of patients with lower DDR scores. However, LIHC patients with low DDR scores showed a significant decrease in the activity of immune depletion and drug resistance-related pathways (**Figure 7B**). The above GSEA results were verified in the ICI-treated BLCA cohort in the same way (**Figure 7C**). We further confirmed the differences in the expression levels of the genes in the above pathways between the low DDR score group and the high DDR score *via* heatmap analysis (**Figures S4**–**S6**).

## Discussion

In this study, we found that there were significant differences in the activation of pathways in different DDR groups in HCC cohorts, and DDR1 patients had low expression of DDR-related genes, while DDR2 patients had high expression of DDR-related genes. After receiving traditional treatment, DDR2 patients had significantly shorter OS than DDR1 patients. In contrast, patients in the DDR2 group had significantly longer OS after receiving ICIs than those in the DDR1 group. After traditional treatment, patients with high DDR scores had worse survival prognoses than those with low DDR scores. However, the survival of patients with high DDR scores after receiving ICIs was significantly higher than that of patients with low DDR scores. The DDR score of patients in the DDR2 group was significantly higher than that of patients in the DDR1 group. To further explore the differences in the TME in different DDR groups, we further explored the potential molecular mechanism underlying the increased response to immunotherapy in the DDR2 group in terms of immune cells, immune-related gene expression, immune signatures and activation of specific pathways. The TME of DDR2 patients has highly infiltrated with activated immune cells and had high expression of immune checkpoint molecules, proinflammatory molecules and antigen presentation-related molecules. GSEA showed that the activity of immune-related pathways, DNA repair pathways and traditional drug resistance pathways in DDR2 patients was significantly higher than that in DDR1 patients, while the activity of some immune depletion pathways in DDR1 patients was significantly higher than that in DDR2 patients. Similarly, the activity of immune pathways, DDR-related pathways and traditional drug resistance pathways was significantly higher in patients with high DDR scores than in those with low DDR scores. Additionally, the CMap algorithm and a heuristic algorithm were used to predict potential drugs for LIHC patients (of the DDR2 type or with a high DDR score).

Patients with DDR1 HCC had lower DDR scores and benefited more from traditional treatment than patients with DDR2 HCC, which may be related to the low DDR activity, cell cycle activity and traditional drug resistance pathway activity in the DDR1 group. Studies have shown that high activity of the MAPK or PI3K/AKT pathway is related to chemotherapy resistance in cancer patients (34). Additionally, when DNA damage occurs, intracellular damage receptors, such as ataxia telangiectasia mutated protein (ATM), Rad3-related protein and the Rad9-Rad1-Hus1 protein complex, detect DNA damage and initiate the signal transduction cascade involving checkpoint kinases 1 and 2 and cell cycle regulators. These cell cycle regulators can block the G1 and S cell cycles stages and the G2/M cell cycle transition by activating p53 and inhibiting cell cycle-dependent kinases, enabling the cells to re-enter the cell cycle after successful repair. Once the DNA in cells cannot be repaired, the apoptosis pathway will be activated in the cells, inducing self-directed apoptosis to prevent the damaged DNA from being transmitted to the offspring (35). Yang et al. found that the overexpression of XRCC4-like factor (XLF), a key gene in the DDR pathway, was significantly related to the poor OS rate of HCC patients receiving traditional treatment. Knocking out XLF increased the sensitivity of HCC to chemotherapy by inhibiting DNA repair (36). Chen et al. showed that reducing the DNA repair ability of HCC cells can further enhance the cytotoxicity of radiotherapy and chemotherapy (11).

Patients with DDR2 cancer had a higher DDR score and benefited less from traditional treatment than patients with DDR1 cancer, but the DDR2 type was significantly related to a longer survival time with immunotherapy. This increased survival in the DDR2 group may be related to the TME of patients in the DDR2 group or patients with a high DDR score, who were more likely to receive immunotherapy. Patients with the DDR2 type had higher proportions of activated immune cells and higher expression of immune checkpoint molecules, chemokines (CXCR3 and CXCL9), proinflammatory factors (such as IFNγ), and antigen processing- and presentation-related molecules (HLA-related molecules) than patients with the DDR1 type. Additionally, the activity of pathways related to immune cell activation, proinflammatory factor secretion, and antigen processing and presentation was significantly higher in DDR2 patients than in DDR1 patients. Immune checkpoint molecules are important targets for ICI therapy, and studies suggest that high expression of immune checkpoint molecules is related to superior immunotherapy efficacy (37, 38). TILs are an important part of HCC TME. High levels of lymphocyte infiltration, especially infiltration of CD4+ T cells and CD8+ T cells, are related to superior prognosis after immunotherapy (39, 40). Andrea Necchi et al. showed that a high lymphocyte infiltration level indicates a strong antitumor immune response across cancers (41). CD8+ T cells are the main TILs in liver cancer and can release perforin and granzyme B through the Fas/FasL pathway or kill target cells by releasing IFN-γ and TNF (42). The expression of Fas/FasL in CD8+ T cells is positively correlated with the antitumor immunity of liver cancer (43). Additionally, cytokines in the TME play an important role in the formation of the inflammatory immune microenvironment (44). For example, chemokines (CXCL9 and CXCR3) can exert antitumor responses by recruiting CD4+ T cells, CD8+ T cells, NK cells and M1 macrophages into the tumor center (45–47). Additionally, IFN-γ can not only promote TILs to exert antitumor reactions but also mediate iron-induced death in tumor cells (48). Additionally, an IFN-γ-related gene expression profile has also been significantly associated with superior ICI efficacy (49). The activity of pathways related to antigen processing and presentation in the TME will also affect the immunogenicity of tumors, and an increase in antigen processing and presentation activity is beneficial for improving the body’s recognition of tumor antigens (50). Additionally, high expression levels of tumor-specific MHC-II molecules are significantly correlated with a superior immunotherapy response (51). Studies have shown that an increase in lipid metabolism can promote cancer metastasis and progression (50). Additionally, the enhancement of cholesterol metabolism will further inhibit T cells from enacting tumor cell killing. Consistent with the published literature (50–52), the activity of some immune exhaustion-related pathways (such as pathways related to cholesterol metabolism and lipid metabolism) was significantly decreased in patients in the DDR2 group.

However, this study also has some limitations. First, due to a lack of an ICI-treated HCC cohort, the differences between the DDR molecular subclasses (DDR1 and DDR2) and the DDR-high and DDR-low score groups could not be further verified. Second, the patients in the TCGA-LIHC and Mongolian-LIHC cohorts may have heterogeneous tumors, which may have a potential impact on the results of this study. We hope to collect and include HCC patients receiving ICI treatment in future research and further verify the influence of DDR molecular type (DDR 1 and DDR2) and the DDR score on the outcomes of HCC patients receiving immunotherapy.

## Conclusions

In this study, through unsupervised clustering of the DDR-related expression profiles of HCC samples, we found that HCC patients could be divided into a DDR1 group (with low activation of DDR pathways) and a DDR2 group (with high activation of DDR pathways). Patients with the DDR2 type had higher DDR scores than those with the DDR1 type. Intriguingly, after traditional treatment, the OS of patients with DDR1 HCC with a low DDR score was significantly prolonged. In contrast, after immunotherapy, DDR2 patients with high DDR scores had a better prognosis than those with low DDR scores. Based on the analysis of the TME, we found that patients with high DDR2 scores had an inflammatory TME, which was characterized by highly enrichment of activated immune cells, high expression of proinflammatory cytokines, high levels of expression of immune signatures and high immune-related pathway activity, while patients with low DDR1 scores had molecular features that are potentially more conducive to response to traditional treatment, such as a low ability to repair DNA damage and low activity of drug resistance pathways functioning in traditional treatment response.

## Data Availability Statement

The original contributions presented in the study are included in the article/**Supplementary Material**. Further inquiries can be directed to the corresponding authors.

## Author Contributions

Conceptualization, KC; Formal analysis, YFC; Visualization, YFC; Writing–original draft, XW, XFD, YZ, RL, YZL and HJY; Writing–review & editing, YFC, XW, XFD, YZ, RL, YZL, HJY and KC. All authors contributed to the article and approved the submitted version.

## Conflict of Interest

The authors declare that the research was conducted in the absence of any commercial or financial relationships that could be construed as a potential conflict of interest.

## Supplementary Material

Supplementary Figure 1 Biological function pathways, such as DNA repair, immune-related, drug resistance and immune exhaustion pathways, identified as enriched between DDR1 and DDR2 tumors in the Mongolian-LIHC cohort. GSEA of hallmark gene sets downloaded from MSigDB. All transcripts were ranked by the log2 (fold change) value between DDR1 and DDR2 tumors. Each run was performed with 1,000 permutations. Pathways with significant enrichment between DDR1 and DDR2 tumors are shown.

Supplementary Figure 2 Heatmap of core genes in significantly enriched pathways between DDR1 and DDR2 tumors in the TCGA-LIHC cohort.

Supplementary Figure 3 Heatmap of core genes in significantly enriched pathways between DDR1 and DDR2 tumors in the Mongolian-LIHC cohort.

Supplementary Figure 4 Heatmap of core genes in significantly enriched pathways between high DDR score and low DDR score tumors in the TCGA-LIHC cohort.

Supplementary Figure 5 Heatmap of core genes in significantly enriched pathways between high DDR score and low DDR score tumors in the Mongolian-LIHC cohort.

Supplementary Figure 6 Heatmap of core genes in significantly differentially enriched pathways between high DDR score and low DDR score tumors in the ICI-treated cohort.
