# Supplementary figures and images for: DNA Damage Repair Status Predicts Opposite Clinical Prognosis Immunotherapy and Non-Immunotherapy in Hepatocellular Carcinoma
Source: Front Immunol. 2021 Jul 15;12:676922. doi: 10.3389/fimmu.2021.676922 (PMC8320764; doi:10.3389/fimmu.2021.676922)

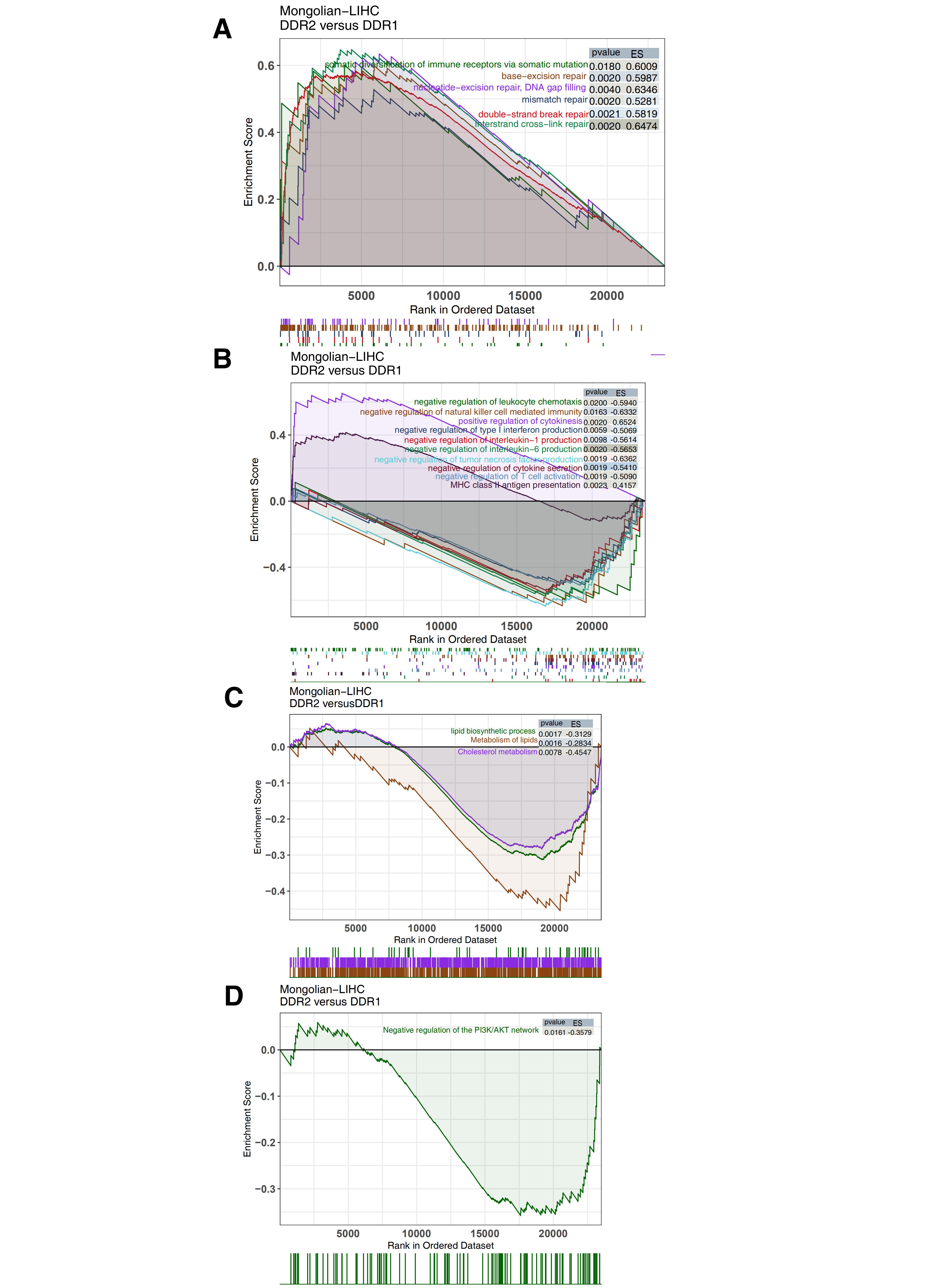

Supplement: Supplementary Figure 1 — Biological function pathways, such as DNA repair, immune-related, drug resistance and immune exhaustion pathways, identified as enriched between DDR1 and DDR2 tumors in the Mongolian-LIHC cohort. GSEA of hallmark gene sets downloaded from MSigDB. All transcripts were ranked by the log2 (fold change) value between DDR1 and DDR2 tumors. Each run was performed with 1,000 permutations. Pathways with significant enrichment between DDR1 and DDR2 tumors are shown. [file Image_1.jpeg]

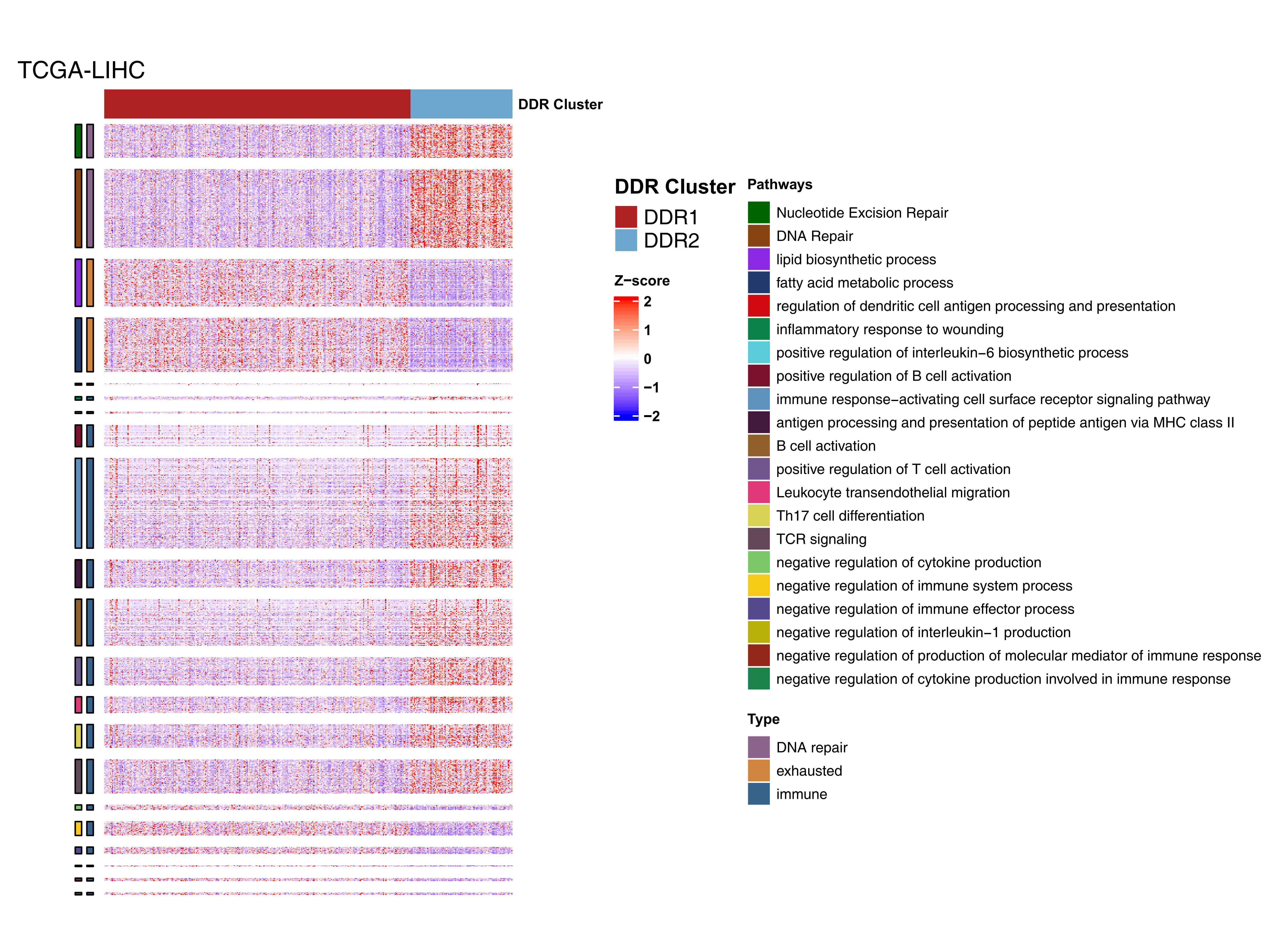

Supplement: Supplementary Figure 2 — Heatmap of core genes in significantly enriched pathways between DDR1 and DDR2 tumors in the TCGA-LIHC cohort. [file Image_2.jpeg]

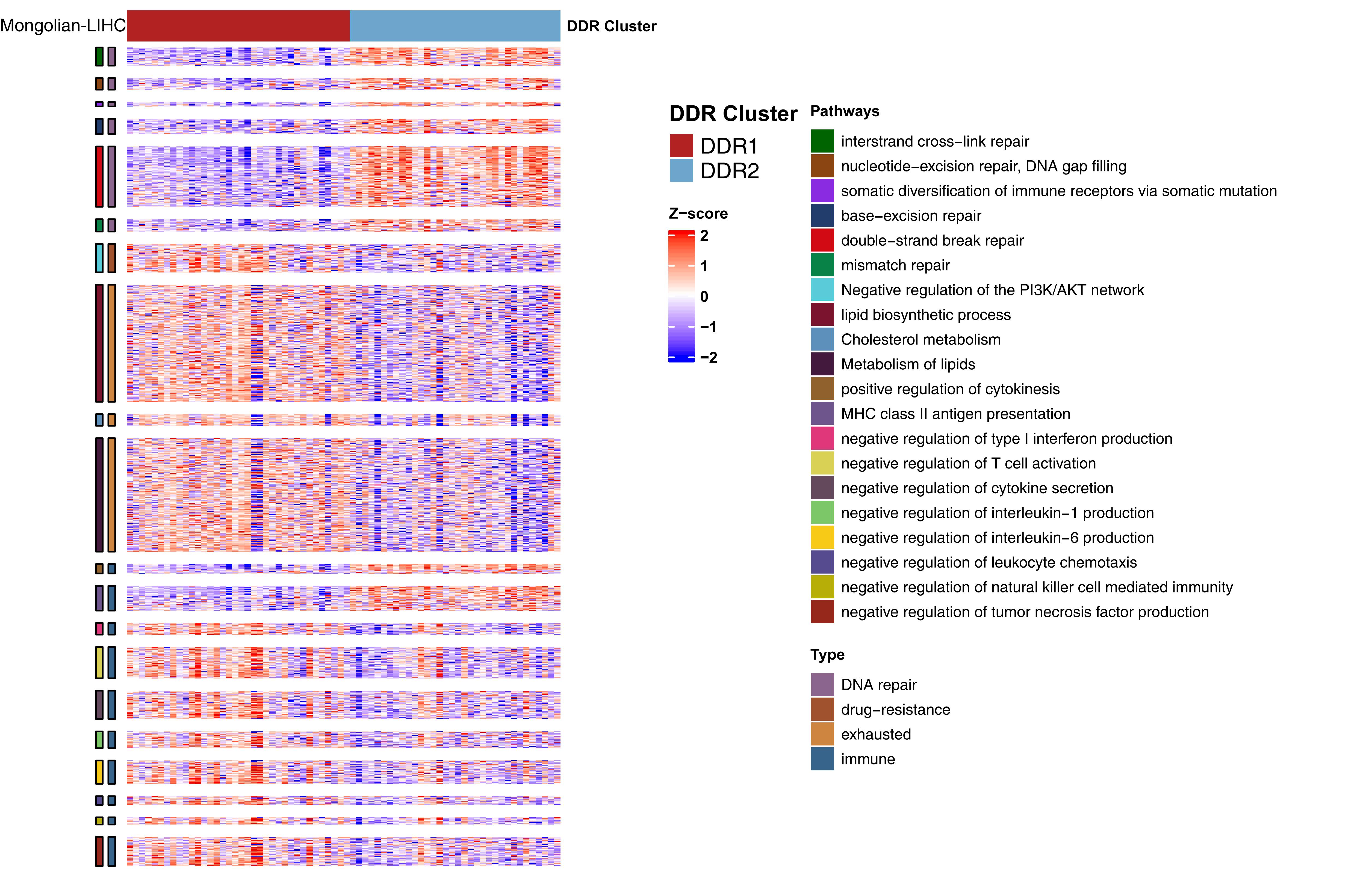

Supplement: Supplementary Figure 3 — Heatmap of core genes in significantly enriched pathways between DDR1 and DDR2 tumors in the Mongolian-LIHC cohort. [file Image_3.jpeg]

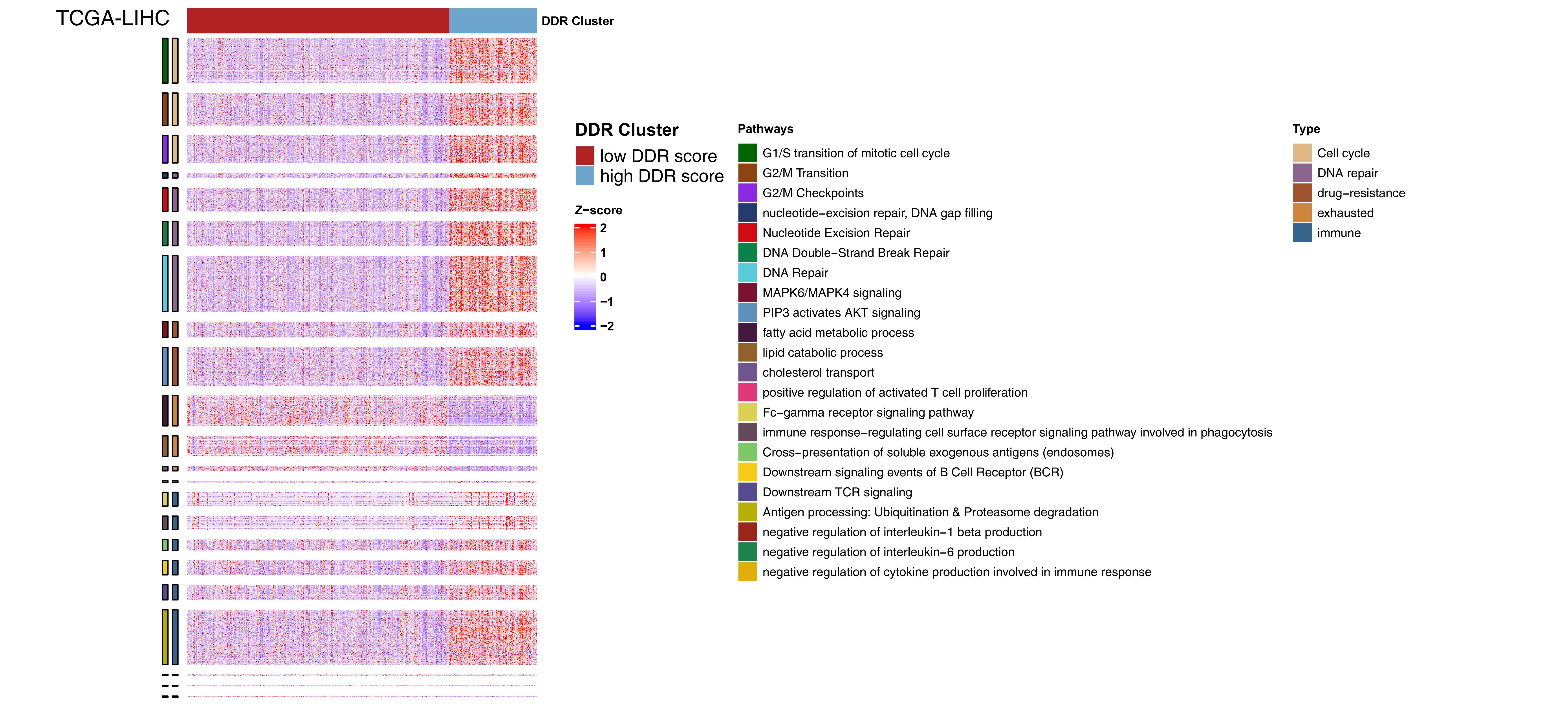

Supplement: Supplementary Figure 4 — Heatmap of core genes in significantly enriched pathways between high DDR score and low DDR score tumors in the TCGA-LIHC cohort. [file Image_4.jpeg]

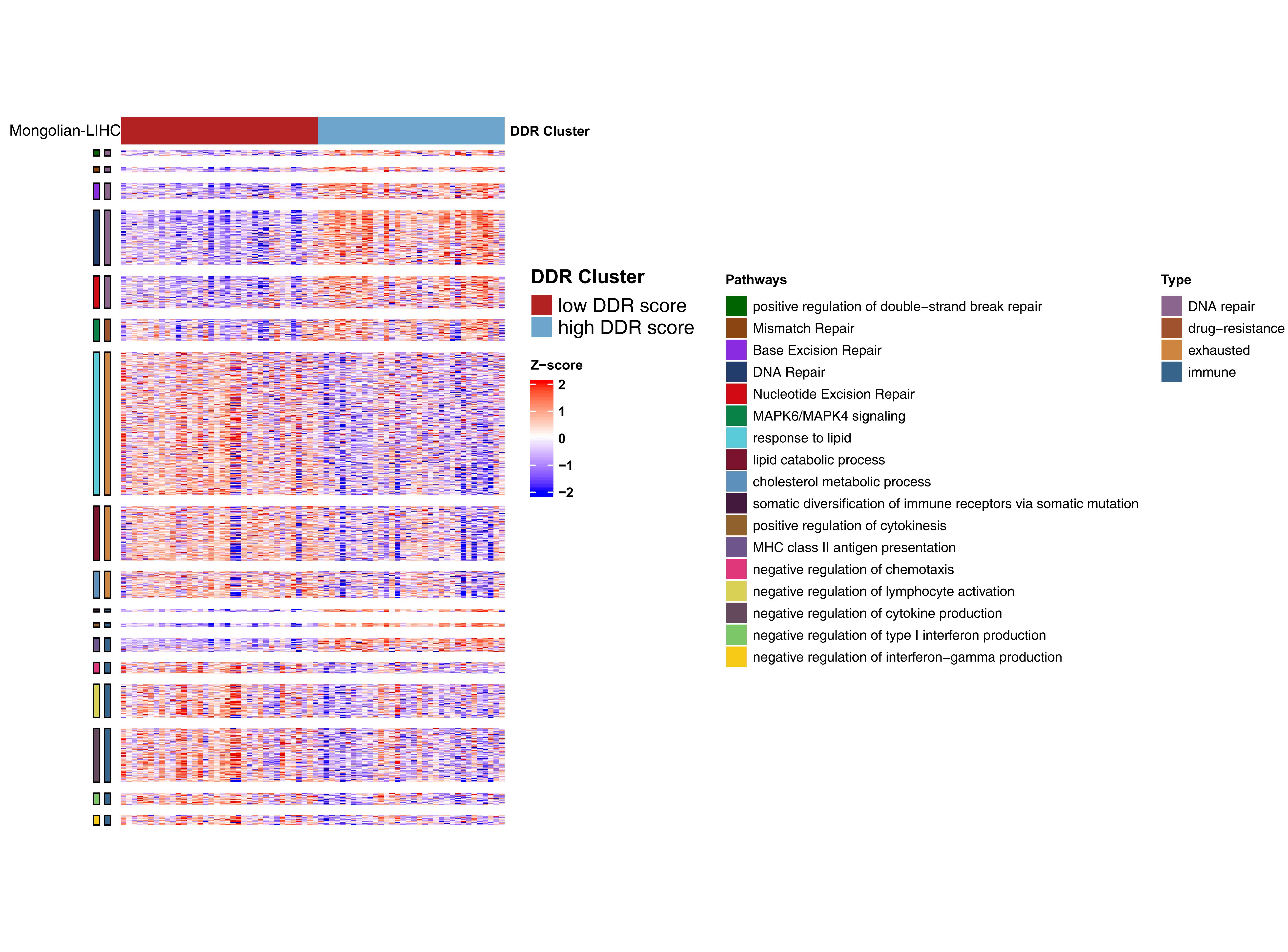

Supplement: Supplementary Figure 5 — Heatmap of core genes in significantly enriched pathways between high DDR score and low DDR score tumors in the Mongolian-LIHC cohort. [file Image_5.jpeg]

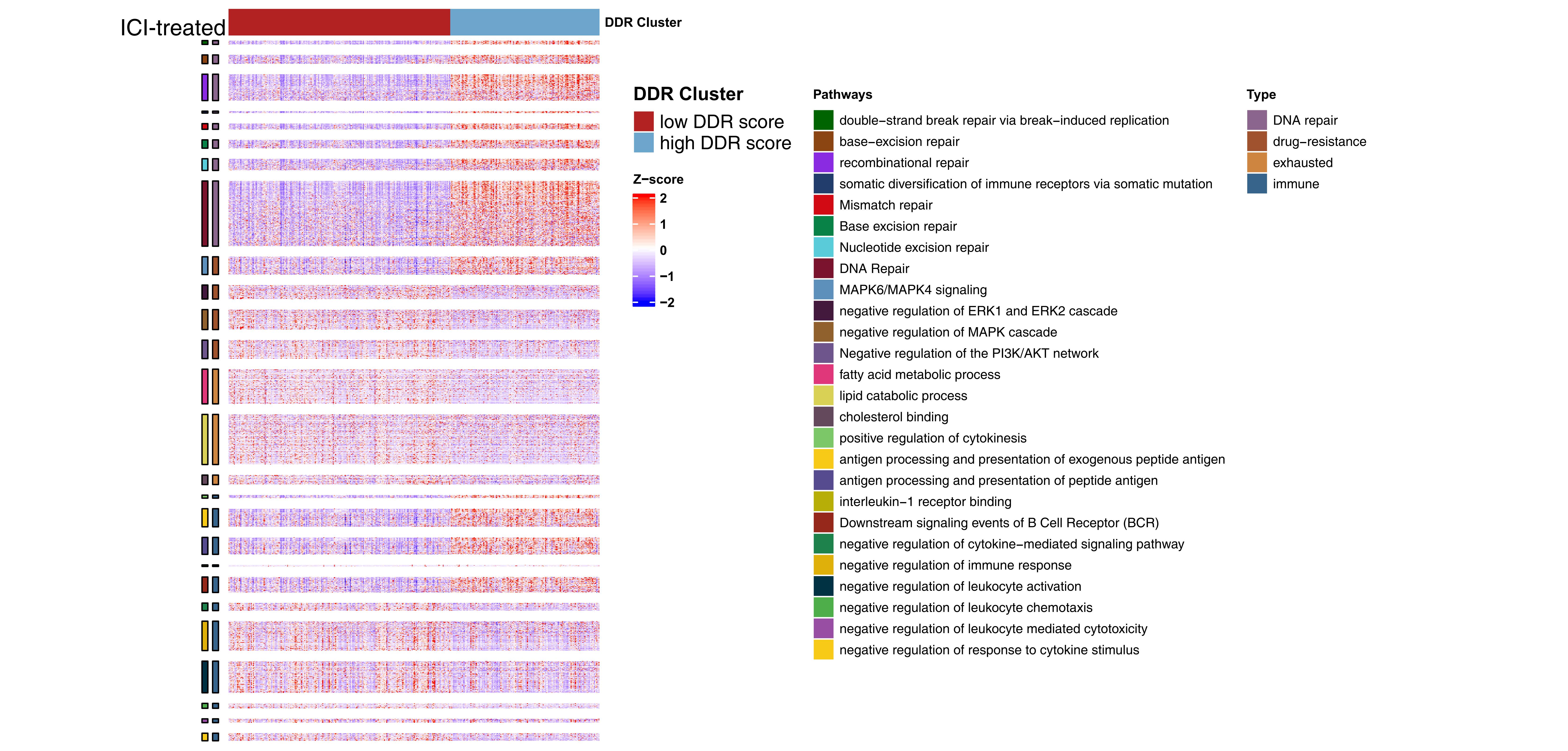

Supplement: Supplementary Figure 6 — Heatmap of core genes in significantly differentially enriched pathways between high DDR score and low DDR score tumors in the ICI-treated cohort. [file Image_6.jpeg]
